# 1,3-Alternate conformer 5,11,17,23-tetra-*tert*-butyl-25,26,27,28-tetra­kis­(4-methyl­sulfanylbenz­yloxy)-2,8,14,20-tetra­thia­calix[4]arene

**DOI:** 10.1107/S1600536813014827

**Published:** 2013-06-08

**Authors:** Qingsong Gao, Dexun Xie, Delie An

**Affiliations:** a Department of Chemistry, College of Chemistry and Chemical Engineering, Hunan University, Changsha 410082, People’s Republic of China

## Abstract

The title thia­calix[4]arene derivative, C_72_H_80_O_4_S_8_, adopts a 1,3-alternate conformation, where the four 4-methyl­sul­fan­yl­benzyl groups are located alternately at the two sides of a virtual plane defined by the four bridging S atoms. In the crystal, there are no significant inter­molecular inter­actions present. Some of the peripheral *tert*-butyl and methyl­sulfanyl groups are disordered over two positions. A region of disordered electron density, occupying voids of *ca* 700 Å^3^ for an electron count of 124, was treated using the SQUEEZE routine in *PLATON* [Spek (2009 ▶). *Acta Cryst*. D**65**, 148–155].

## Related literature

For a similar compound adopting a 1,3-alternate conformation, see: Xu *et al.* (2008 ▶). For background to thia­calix[4]arene derivatives, see: Kumagai *et al.* (1997 ▶); Morohashi *et al.* (2006 ▶); Yamato *et al.* (2006 ▶). For background to multidentate methyl­thio­ethers, see: Maye *et al.* (2005 ▶); Lim *et al.* (2007 ▶); Yan *et al.* (2010 ▶). For the synthesis, see: Morohashi *et al.* (2003 ▶).
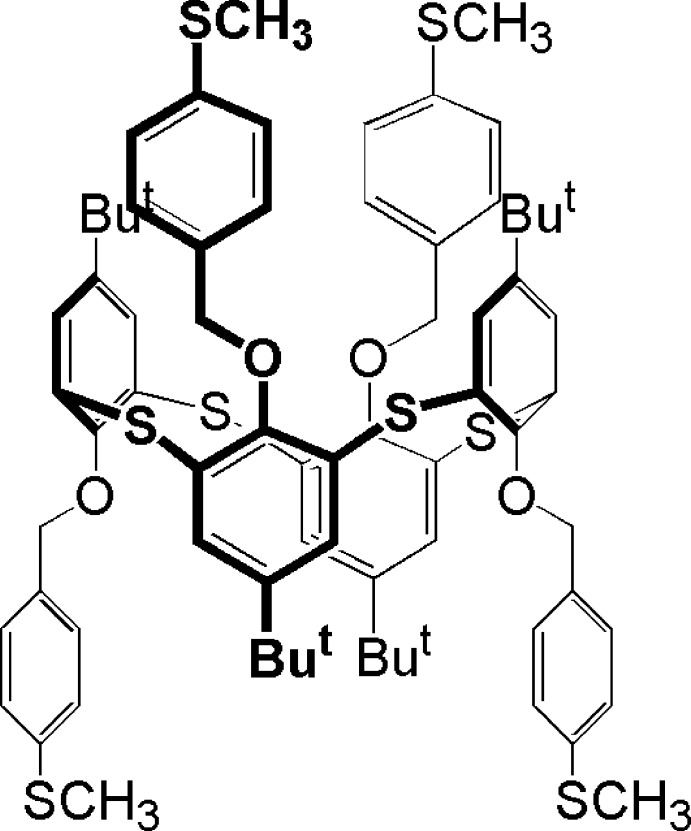


## Experimental

### 

#### Crystal data

C_72_H_80_O_4_S_8_
*M* *_r_* = 1265.84
Triclinic, 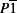

*a* = 15.1863 (10) Å
*b* = 15.5795 (11) Å
*c* = 16.9774 (12) Å
α = 75.473 (2)°
β = 85.686 (2)°
γ = 84.762 (2)°
*V* = 3866.4 (5) Å^3^
*Z* = 2
Mo *K*α radiation
μ = 0.27 mm^−1^
*T* = 293 K
0.26 × 0.21 × 0.15 mm

#### Data collection

Bruker SMART CCD area-detector diffractometer
Absorption correction: multi-scan (*SADABS*; Bruker, 2007 ▶) *T*_min_ = 0.434, *T*_max_ = 1.000
14394 measured reflections
14394 independent reflections
9034 reflections with *I* > 2σ(*I*)

#### Refinement

*R*[*F* ^2^ > 2σ(*F* ^2^)] = 0.059
*wR*(*F* ^2^) = 0.172
*S* = 0.96
14394 reflections
939 parameters
244 restraints
H-atom parameters constrained
Δρ_max_ = 0.40 e Å^−3^
Δρ_min_ = −0.29 e Å^−3^

### 

Data collection: *SMART* (Bruker, 2007 ▶); cell refinement: *SAINT* (Bruker, 2007 ▶); data reduction: *SAINT*; program(s) used to solve structure: *SHELXS97* (Sheldrick, 2008 ▶); program(s) used to refine structure: *SHELXL97* (Sheldrick, 2008 ▶); molecular graphics: *SHELXTL* (Sheldrick, 2008 ▶); software used to prepare material for publication: *SHELXTL*.

## Supplementary Material

Crystal structure: contains datablock(s) I, global. DOI: 10.1107/S1600536813014827/su2608sup1.cif

Structure factors: contains datablock(s) I. DOI: 10.1107/S1600536813014827/su2608Isup2.hkl

Additional supplementary materials:  crystallographic information; 3D view; checkCIF report

## Figures and Tables

**Fig. 1.**
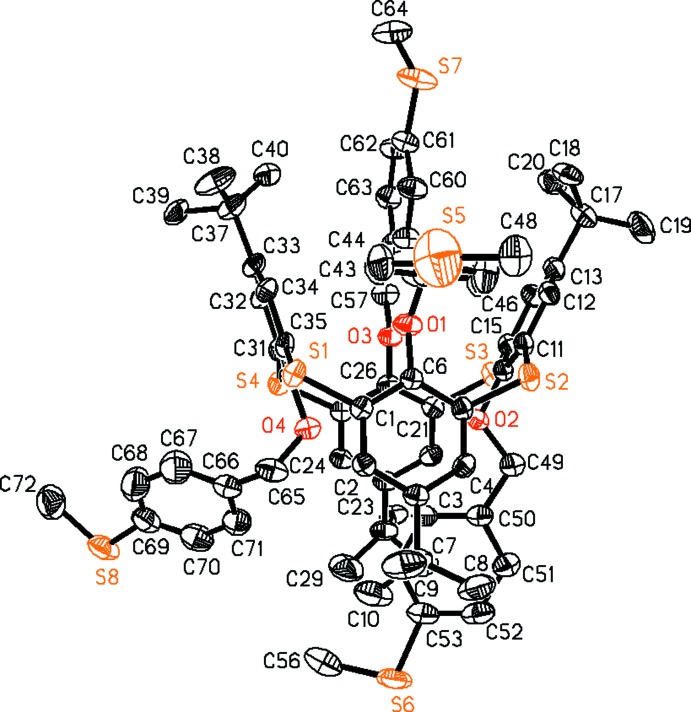
A view of the molecular structure of the title compound, with atom labelling. Displacement ellipsoids are drawn at the 50% probability level. Only the major components of the disordered peripheral groups are shown.

## supplementary crystallographic information

### Comment

Thiacalix[4]arenes are macrocyclic molecules made up of *p*-substituted phenolic units linked by sulfur atoms *ortho* to the OH functions (Kumagai *et al.*, 1997). The ability of the parent phenolic thiacalix[4]arenes, as well as of their chemically modified derivatives obtained by the substitution of the phenolic H atoms with various types of ligating groups, to bind metal ions is well established (Morohashi *et al*., 2006). With thiacalix[4]arenes, the substituents frequently immobilize the molecule in a single conformation: cone, partial cone, 1,2- or 1,3-alternate. The ability to control inter-particle spatial properties of nanoparticle assemblies is one of the major challenges for the design and understanding of functional nanostructures. As a molecular linker, multidentate thioethers have been exploited for such control (Maye *et al*., 2005). The viability of inter-particle linkages *via* coordination of the methylthio groups of arylethynes to gold surfaces was demonstrated recently in our laboratory (Lim *et al.*, 2007; Yan *et al.*, 2010). Multi-functional groups is the common characteristic of these molecular linkers. The 1,3-alternate conformer thiacalixarene derivative is an ideal molecular linker for assembling nanoparticle clusters. With this in mind, we synthesized the title compound, the first example of a thiacalix[4]arene derivative containing multidentate methyithioethers, and we report herein on its crystal structure.

The molecular structure of the title molecule is shown in Fig 1. The macrocycle adopts a 1,3-alternate conformation in which four substituent groups are located alternately above and below the virtual plane defined by four bridging sulfur atoms, S1-S4. The 1,3-alternate conformation thus appears to be regular and two pairs of opposite phenolic units are almost parallel to each other, but the substituent groups are inclined to one another. Comparable conformations were found in methyl ester derivatives (Xu *et al.*, 2008), whereas the title tetra-benzyl ether derivative is much more distorted as a result of increased steric hindrance.

The plane defined by the substitutional aromatic ring on O4 atom (r.m.s. deviation 0.0177 Å) was chosen as a reference plane. The plane defined by the other substitutional aromatic rings on O atoms (O1, O2 and O3) make dihedral angles of 87.83 (11), 76.84 (11) and 71.78 (13) °, respectively, with this reference plane, whereas the four aromatic rings on the skeleton make dihedral angles of 83.52 (8), 76.20 (8), 84.47 (8) and 83.09 (8) °, respectively, with this reference plane. The conformations of the benzyl ether chains are extended and deviate from the plane defined by four bridging sulfur atoms. Atoms C41, C49, C57 and C65 point towards the exterior of the macrocycle and the torsion angles around the O1—C41, C49—O2, C57—O3 and C65—O4 bonds deviate from ideal *syn* or *anti* values by more than 70°.

In the crystal, there are no significant intermolecular interactions present.

### Experimental

A mixture of *p*-tetra-*tert*-butylthiacalix[4]arene (360 mg, 0.50 mmol) and Cs_2_CO_3_ (1.30 g, 4.00 mmol) in anhydrous acetone (50 ml) was heated at refluxed for 30 min. Then a solution of 4-methylthiobenzyl bromide (864 mg, 4.00 mmol) in acetone (10 ml) was added and the mixture heated at reflux for 2 h. After cooling the reaction mixture, it was filtered. The filtrate was concentrated and the residue was purified by column chromatography from petroleum ether/dichloromethane (4:1, *v*/*v*) to give 410 mg (65%) of compound I as a white solid: M.p. 513~516 K; MS(ESI) m/*z*: 1283.1 [*M*+H_2_O]^+^. Spectroscopic data for the title compound is available in the archived CIF. Colourless crystals of the title compound, suitable for X-ray diffraction analysis, were obtained by slow diffusion of petroleum ether into a chloroform solution at 298 K.

### Refinement

Some of the peripheral -SCH_3_ and *t*-butyl groups are disordered over two positions. These include the S-CH_3_ groups involving atoms S5-C48, S6-C56, S7-C64 and S8-C72, and the *t*-butyl groups involving atoms C18-C20, C28-C30 and C38-C40; details are available in the archived CIF. A region of disordered electron density occupying voids of ca. 700 Å^3^, for an electron count of 124, was treated using the SQUEEZE routine in PLATON (Spek, 2009). It was not taken into consideration during refinement. The C-bound H atoms were positioned geometrically and allowed to ride on their parent atoms: C—H = 0.93–0.97 Å with *U*_iso_(H) = 1.5U_eq_(C) for methyl H atoms and = 1.2U_eq_(C) for other H atoms.

### Crystal data

**Table:**

C_72_H_80_O_4_S_8_	*Z* = 2
*M**_r_* = 1265.84	*F*(000) = 1344
Triclinic, *P*1	*D*_x_ = 1.087 Mg m^−^^3^
Hall symbol: -P 1	Mo *K*α radiation, λ = 0.71073 Å
*a* = 15.1863 (10) Å	Cell parameters from 4356 reflections
*b* = 15.5795 (11) Å	θ = 4.4–45.1°
*c* = 16.9774 (12) Å	µ = 0.27 mm^−^^1^
α = 75.473 (2)°	*T* = 293 K
β = 85.686 (2)°	Prismatic, colourless
γ = 84.762 (2)°	0.26 × 0.21 × 0.15 mm
*V* = 3866.4 (5) Å^3^	

### Data collection

**Table:**

Bruker SMART CCD area-detector diffractometer	14394 independent reflections
Radiation source: fine-focus sealed tube	9034 reflections with *I* > 2σ(*I*)
Graphite monochromator	*R*_int_ = 0.000
phi and ω scans	θ_max_ = 25.5°, θ_min_ = 1.4°
Absorption correction: multi-scan (*SADABS*; Bruker, 2007)	*h* = −18→18
*T*_min_ = 0.434, *T*_max_ = 1.000	*k* = −18→18
14394 measured reflections	*l* = 0→20

### Refinement

**Table:**

Refinement on *F*^2^	Primary atom site location: structure-invariant direct methods
Least-squares matrix: full	Secondary atom site location: difference Fourier map
*R*[*F*^2^ > 2σ(*F*^2^)] = 0.059	Hydrogen site location: inferred from neighbouring sites
*wR*(*F*^2^) = 0.172	H-atom parameters constrained
*S* = 0.96	*w* = 1/[σ^2^(*F*_o_^2^) + (0.1009*P*)^2^] where *P* = (*F*_o_^2^ + 2*F*_c_^2^)/3
14394 reflections	(Δ/σ)_max_ = 0.004
939 parameters	Δρ_max_ = 0.40 e Å^−^^3^
244 restraints	Δρ_min_ = −0.29 e Å^−^^3^

### Special details

**Table:**

Experimental. Spectroscopic data for the title compound:^1^H NMR (400 MHz, CDCl_3_): 7.12 (s, 8H), 7.05 (d, J = 8.4 Hz, 8H), 6.92 (d, J = 8.4 Hz, 8H), 5.04 (s, 8H), 2.48 (s, 12H, SCH_3_), 0.86 (s, 36H); ^13^C NMR (100 MHz, CDCl_3_): 156.89 (C), 146.15 (C), 136.67 (C), 134.87 (C), 129.26 (CH), 128.57 (C), 127.69 (CH), 126.95 (CH), 70.65 (CH_2_), 33.86 (C), 30.73 (CH_3_), 16.31 (SCH_3_).
Geometry. All e.s.d.'s (except the e.s.d. in the dihedral angle between two l.s. planes) are estimated using the full covariance matrix. The cell e.s.d.'s are taken into account individually in the estimation of e.s.d.'s in distances, angles and torsion angles; correlations between e.s.d.'s in cell parameters are only used when they are defined by crystal symmetry. An approximate (isotropic) treatment of cell e.s.d.'s is used for estimating e.s.d.'s involving l.s. planes.
Refinement. Refinement of *F*^2^ against ALL reflections. The weighted *R*-factor *wR* and goodness of fit *S* are based on *F*^2^, conventional *R*-factors *R* are based on *F*, with *F* set to zero for negative *F*^2^. The threshold expression of *F*^2^ > σ(*F*^2^) is used only for calculating *R*-factors(gt) *etc*. and is not relevant to the choice of reflections for refinement. *R*-factors based on *F*^2^ are statistically about twice as large as those based on *F*, and *R*- factors based on ALL data will be even larger.

### Fractional atomic coordinates and isotropic or equivalent isotropic displacement parameters (Å^2^)

**Table:**

	*x*	*y*	*z*	*U*_iso_*/*U*_eq_	Occ. (<1)
S1	0.94198 (4)	0.90174 (4)	0.09861 (4)	0.05036 (19)	
S2	0.92245 (5)	0.55348 (5)	0.07790 (5)	0.0582 (2)	
S3	0.55404 (5)	0.59267 (5)	0.09846 (5)	0.0631 (2)	
S4	0.57286 (4)	0.93527 (5)	0.13515 (4)	0.0541 (2)	
S5	1.3408 (4)	0.7583 (4)	−0.0886 (7)	0.1079 (15)	0.70
S6	0.7412 (5)	0.5926 (7)	0.5541 (5)	0.1038 (18)	0.50
S7	0.7645 (7)	0.8754 (7)	−0.3436 (5)	0.101 (2)	0.50
S8	0.5403 (5)	1.1277 (5)	0.4157 (3)	0.0949 (13)	0.55
C48	1.3875 (3)	0.6516 (4)	−0.0944 (4)	0.0867 (19)	0.65
H48A	1.3728	0.6089	−0.0448	0.130*	0.65
H48B	1.4507	0.6526	−0.1023	0.130*	0.65
H48C	1.3644	0.6355	−0.1394	0.130*	0.65
C56	0.7113 (9)	0.7107 (9)	0.5414 (10)	0.122 (4)	0.50
H56A	0.7601	0.7438	0.5151	0.184*	0.50
H56B	0.6971	0.7227	0.5939	0.184*	0.50
H56C	0.6608	0.7279	0.5087	0.184*	0.50
C64	0.6894 (8)	0.9425 (8)	−0.4157 (6)	0.118 (4)	0.50
H64A	0.6667	0.9941	−0.3974	0.177*	0.50
H64B	0.6413	0.9084	−0.4203	0.177*	0.50
H64C	0.7203	0.9606	−0.4679	0.177*	0.50
C72	0.5345 (9)	1.2383 (7)	0.3516 (8)	0.119 (3)	0.60
H72D	0.5930	1.2584	0.3398	0.179*	0.60
H72E	0.4987	1.2772	0.3789	0.179*	0.60
H72F	0.5086	1.2385	0.3016	0.179*	0.60
S5'	1.3463 (11)	0.7406 (12)	−0.0752 (17)	0.1084 (16)	0.30
S6'	0.7109 (6)	0.6042 (8)	0.5530 (6)	0.122 (3)	0.50
S7'	0.7480 (8)	0.8905 (8)	−0.3466 (5)	0.117 (3)	0.50
S8'	0.5571 (8)	1.1460 (8)	0.4075 (6)	0.140 (4)	0.45
C48'	1.3332 (13)	0.6556 (10)	−0.1267 (10)	0.138 (5)	0.35
H48D	1.3409	0.5983	−0.0894	0.207*	0.35
H48E	1.3766	0.6597	−0.1714	0.207*	0.35
H48F	1.2749	0.6635	−0.1470	0.207*	0.35
C56'	0.7588 (10)	0.7043 (10)	0.5410 (10)	0.138 (5)	0.50
H56D	0.7428	0.7439	0.4901	0.208*	0.50
H56E	0.8220	0.6935	0.5412	0.208*	0.50
H56F	0.7379	0.7307	0.5850	0.208*	0.50
C64'	0.6973 (10)	0.9922 (8)	−0.4005 (8)	0.143 (5)	0.50
H64D	0.6341	0.9911	−0.3922	0.215*	0.50
H64E	0.7136	1.0011	−0.4576	0.215*	0.50
H64F	0.7164	1.0399	−0.3812	0.215*	0.50
C72'	0.5716 (15)	1.2563 (14)	0.3432 (17)	0.174 (11)	0.40
H72A	0.6305	1.2720	0.3472	0.260*	0.40
H72B	0.5294	1.2984	0.3607	0.260*	0.40
H72C	0.5627	1.2568	0.2877	0.260*	0.40
O1	0.89430 (12)	0.76024 (13)	0.01849 (11)	0.0584 (5)	
O2	0.74105 (12)	0.60108 (12)	0.15708 (10)	0.0515 (4)	
O3	0.61701 (11)	0.78218 (12)	0.04810 (10)	0.0482 (4)	
O4	0.75495 (12)	0.85942 (12)	0.18907 (11)	0.0554 (5)	
C1	0.94462 (15)	0.78558 (16)	0.14019 (15)	0.0422 (6)	
C2	0.97394 (16)	0.75315 (17)	0.21775 (15)	0.0475 (6)	
H2	0.9865	0.7933	0.2470	0.057*	
C3	0.98546 (16)	0.66224 (18)	0.25391 (15)	0.0478 (6)	
C4	0.96376 (16)	0.60604 (17)	0.20888 (16)	0.0486 (6)	
H4	0.9686	0.5451	0.2319	0.058*	
C5	0.93484 (15)	0.63659 (16)	0.13047 (15)	0.0433 (6)	
C6	0.92664 (15)	0.72676 (16)	0.09419 (14)	0.0410 (6)	
C7	1.01959 (18)	0.6293 (2)	0.34044 (16)	0.0604 (8)	
C8	1.0129 (3)	0.5297 (3)	0.3732 (2)	0.1042 (13)	
H8A	0.9520	0.5165	0.3766	0.156*	
H8B	1.0361	0.5110	0.4264	0.156*	
H8C	1.0465	0.4987	0.3372	0.156*	
C9	1.1168 (2)	0.6489 (3)	0.3363 (2)	0.1014 (13)	
H9A	1.1506	0.6166	0.3016	0.152*	
H9B	1.1387	0.6309	0.3900	0.152*	
H9C	1.1220	0.7115	0.3148	0.152*	
C10	0.9657 (3)	0.6748 (3)	0.3990 (2)	0.1032 (13)	
H10A	0.9707	0.7378	0.3814	0.155*	
H10B	0.9876	0.6529	0.4526	0.155*	
H10C	0.9048	0.6625	0.4004	0.155*	
C11	0.81713 (17)	0.57302 (16)	0.03584 (16)	0.0468 (6)	
C12	0.81386 (18)	0.56067 (17)	−0.04176 (16)	0.0513 (7)	
H12	0.8667	0.5513	−0.0709	0.062*	
C13	0.73439 (19)	0.56175 (18)	−0.07774 (16)	0.0536 (7)	
C14	0.65774 (18)	0.57739 (19)	−0.03304 (16)	0.0545 (7)	
H14	0.6035	0.5799	−0.0559	0.065*	
C15	0.65889 (17)	0.58959 (17)	0.04548 (16)	0.0494 (6)	
C16	0.73887 (17)	0.58747 (16)	0.08053 (15)	0.0455 (6)	
C17	0.7309 (2)	0.5441 (2)	−0.16228 (19)	0.0693 (9)	
C18	0.8077 (14)	0.5946 (9)	−0.2193 (10)	0.079 (3)	0.50
H18A	0.8642	0.5692	−0.1993	0.119*	0.50
H18B	0.8012	0.6565	−0.2193	0.119*	0.50
H18C	0.8042	0.5885	−0.2739	0.119*	0.50
C19	0.7130 (6)	0.4463 (7)	−0.1505 (7)	0.099 (3)	0.60
H19A	0.7058	0.4348	−0.2026	0.148*	0.60
H19B	0.6599	0.4340	−0.1166	0.148*	0.60
H19C	0.7619	0.4089	−0.1251	0.148*	0.60
C20	0.6510 (10)	0.6081 (7)	−0.2097 (9)	0.081 (3)	0.50
H20A	0.6626	0.6692	−0.2167	0.121*	0.50
H20B	0.5962	0.5966	−0.1784	0.121*	0.50
H20C	0.6471	0.5962	−0.2621	0.121*	0.50
C18'	0.8117 (15)	0.5609 (9)	−0.2147 (12)	0.095 (5)	0.50
H18D	0.8041	0.5475	−0.2659	0.143*	0.50
H18E	0.8606	0.5239	−0.1887	0.143*	0.50
H18F	0.8235	0.6222	−0.2240	0.143*	0.50
C19'	0.7610 (10)	0.4428 (12)	−0.1482 (11)	0.112 (5)	0.40
H19D	0.7581	0.4249	−0.1981	0.169*	0.40
H19E	0.7226	0.4089	−0.1068	0.169*	0.40
H19F	0.8207	0.4325	−0.1312	0.169*	0.40
C20'	0.6462 (10)	0.5704 (7)	−0.1996 (9)	0.087 (3)	0.50
H20D	0.6358	0.6339	−0.2119	0.131*	0.50
H20E	0.5997	0.5438	−0.1625	0.131*	0.50
H20F	0.6471	0.5507	−0.2489	0.131*	0.50
C21	0.55120 (16)	0.67324 (18)	0.15619 (15)	0.0480 (6)	
C22	0.51071 (17)	0.64880 (19)	0.23451 (16)	0.0524 (7)	
H22	0.4942	0.5910	0.2547	0.063*	
C23	0.49475 (16)	0.70917 (19)	0.28255 (15)	0.0506 (7)	
C24	0.52130 (16)	0.79369 (19)	0.25020 (15)	0.0485 (6)	
H24	0.5121	0.8350	0.2817	0.058*	
C25	0.56121 (15)	0.81970 (17)	0.17237 (15)	0.0448 (6)	
C26	0.57623 (15)	0.75879 (17)	0.12452 (14)	0.0432 (6)	
C27	0.4468 (2)	0.6825 (2)	0.36633 (17)	0.0648 (8)	
C28	0.4834 (8)	0.5980 (8)	0.4150 (7)	0.078 (3)	0.55
H28A	0.4491	0.5821	0.4657	0.118*	0.55
H28B	0.5436	0.6033	0.4257	0.118*	0.55
H28C	0.4817	0.5528	0.3859	0.118*	0.55
C29	0.4863 (8)	0.7236 (9)	0.4259 (8)	0.120 (4)	0.50
H29A	0.4622	0.6988	0.4800	0.180*	0.50
H29B	0.4723	0.7867	0.4117	0.180*	0.50
H29C	0.5494	0.7113	0.4240	0.180*	0.50
C30	0.3469 (7)	0.6736 (6)	0.3514 (6)	0.063 (2)	0.55
H30A	0.3438	0.6265	0.3247	0.095*	0.55
H30B	0.3228	0.7284	0.3176	0.095*	0.55
H30C	0.3135	0.6604	0.4027	0.095*	0.55
C28'	0.4615 (12)	0.5716 (11)	0.4032 (12)	0.107 (6)	0.45
H28D	0.5218	0.5519	0.3921	0.161*	0.45
H28E	0.4227	0.5428	0.3776	0.161*	0.45
H28F	0.4482	0.5569	0.4610	0.161*	0.45
C29'	0.4421 (7)	0.7589 (7)	0.4124 (7)	0.096 (3)	0.50
H29D	0.4133	0.7393	0.4654	0.144*	0.50
H29E	0.4092	0.8105	0.3818	0.144*	0.50
H29F	0.5010	0.7735	0.4180	0.144*	0.50
C30'	0.3535 (11)	0.7105 (9)	0.3632 (10)	0.100 (5)	0.45
H30D	0.3283	0.6887	0.3225	0.150*	0.45
H30E	0.3459	0.7743	0.3498	0.150*	0.45
H30F	0.3242	0.6872	0.4154	0.150*	0.45
C31	0.67539 (16)	0.94933 (16)	0.07791 (16)	0.0457 (6)	
C32	0.67360 (17)	1.00449 (17)	0.00043 (17)	0.0520 (7)	
H32	0.6191	1.0264	−0.0209	0.062*	
C33	0.75021 (17)	1.02849 (17)	−0.04684 (16)	0.0506 (7)	
C34	0.82966 (16)	0.99078 (16)	−0.01344 (16)	0.0465 (6)	
H34	0.8823	1.0034	−0.0443	0.056*	
C35	0.83338 (15)	0.93503 (15)	0.06423 (16)	0.0424 (6)	
C36	0.75657 (16)	0.91547 (15)	0.11223 (15)	0.0424 (6)	
C37	0.7462 (2)	1.0937 (2)	−0.13094 (19)	0.0690 (9)	
C38	0.8285 (5)	1.1254 (7)	−0.1654 (6)	0.100 (3)	0.50
H38A	0.8696	1.0759	−0.1697	0.151*	0.50
H38B	0.8513	1.1579	−0.1313	0.151*	0.50
H38C	0.8203	1.1637	−0.2187	0.151*	0.50
C39	0.6820 (5)	1.1826 (4)	−0.1164 (4)	0.0743 (19)	0.50
H39A	0.6760	1.2258	−0.1675	0.112*	0.50
H39B	0.7086	1.2082	−0.0790	0.112*	0.50
H39C	0.6245	1.1645	−0.0944	0.112*	0.50
C40	0.6885 (7)	1.0612 (6)	−0.1841 (6)	0.077 (3)	0.60
H40A	0.6291	1.0590	−0.1605	0.116*	0.60
H40B	0.7109	1.0028	−0.1884	0.116*	0.60
H40C	0.6888	1.1010	−0.2374	0.116*	0.60
C38'	0.8304 (6)	1.0565 (6)	−0.1917 (4)	0.092 (2)	0.50
H38D	0.8247	0.9951	−0.1898	0.138*	0.50
H38E	0.8867	1.0626	−0.1722	0.138*	0.50
H38F	0.8266	1.0912	−0.2469	0.138*	0.50
C39'	0.7743 (8)	1.1786 (5)	−0.1287 (5)	0.110 (3)	0.50
H39D	0.7749	1.2171	−0.1826	0.166*	0.50
H39E	0.8327	1.1709	−0.1087	0.166*	0.50
H39F	0.7339	1.2046	−0.0933	0.166*	0.50
C40'	0.6673 (10)	1.0938 (10)	−0.1712 (10)	0.093 (5)	0.40
H40D	0.6777	1.1173	−0.2288	0.140*	0.40
H40E	0.6210	1.1301	−0.1509	0.140*	0.40
H40F	0.6500	1.0341	−0.1611	0.140*	0.40
C41	0.9429 (2)	0.7617 (3)	−0.05348 (19)	0.0923 (12)	
H41A	0.9234	0.8159	−0.0924	0.111*	
H41B	0.9263	0.7125	−0.0733	0.111*	
C42	1.04093 (19)	0.7569 (2)	−0.05638 (16)	0.0562 (7)	
C43	1.0881 (2)	0.8287 (2)	−0.0609 (2)	0.0734 (9)	
H43	1.0571	0.8819	−0.0570	0.088*	
C44	1.1777 (2)	0.8264 (2)	−0.0708 (2)	0.0770 (9)	
H44	1.2062	0.8780	−0.0755	0.092*	
C45	1.2255 (2)	0.7502 (2)	−0.07393 (19)	0.0663 (8)	
C46	1.1828 (2)	0.6758 (2)	−0.0680 (2)	0.0847 (11)	
H46	1.2149	0.6226	−0.0702	0.102*	
C47	1.0911 (2)	0.6795 (2)	−0.0585 (2)	0.0836 (10)	
H47	1.0627	0.6278	−0.0534	0.100*	
C49	0.7374 (2)	0.5227 (2)	0.22128 (18)	0.0722 (9)	
H49A	0.7884	0.4819	0.2158	0.087*	
H49B	0.6844	0.4935	0.2185	0.087*	
C50	0.73654 (19)	0.5463 (2)	0.30111 (16)	0.0590 (7)	
C51	0.7421 (2)	0.4787 (2)	0.3715 (2)	0.0797 (10)	
H51	0.7469	0.4199	0.3679	0.096*	
C52	0.7408 (3)	0.4970 (3)	0.4467 (2)	0.0885 (11)	
H52	0.7463	0.4504	0.4929	0.106*	
C53	0.7316 (2)	0.5822 (3)	0.45457 (18)	0.0762 (10)	
C54	0.7265 (2)	0.6505 (2)	0.38446 (18)	0.0738 (9)	
H54	0.7215	0.7093	0.3884	0.089*	
C55	0.7288 (2)	0.6322 (2)	0.30871 (17)	0.0635 (8)	
H55	0.7250	0.6789	0.2623	0.076*	
C57	0.55681 (18)	0.8144 (2)	−0.01567 (16)	0.0621 (8)	
H57A	0.5263	0.8702	−0.0101	0.074*	
H57B	0.5129	0.7718	−0.0119	0.074*	
C58	0.60755 (17)	0.82777 (18)	−0.09687 (15)	0.0498 (6)	
C59	0.69751 (18)	0.8091 (2)	−0.10537 (17)	0.0595 (8)	
H59	0.7297	0.7851	−0.0594	0.071*	
C60	0.7401 (2)	0.8257 (2)	−0.18123 (18)	0.0677 (9)	
H60	0.8009	0.8121	−0.1860	0.081*	
C61	0.6942 (2)	0.8625 (2)	−0.25093 (17)	0.0671 (8)	
C62	0.6036 (2)	0.8789 (2)	−0.24219 (18)	0.0712 (9)	
H62	0.5710	0.9016	−0.2881	0.085*	
C63	0.56160 (19)	0.8621 (2)	−0.16682 (18)	0.0613 (8)	
H63	0.5005	0.8739	−0.1621	0.074*	
C65	0.7901 (2)	0.8914 (2)	0.25194 (18)	0.0726 (9)	
H65A	0.8429	0.9217	0.2296	0.087*	
H65B	0.8078	0.8408	0.2955	0.087*	
C66	0.7271 (2)	0.9535 (2)	0.28716 (18)	0.0674 (8)	
C67	0.7308 (4)	1.0429 (3)	0.2639 (3)	0.1251 (17)	
H67	0.7725	1.0668	0.2233	0.150*	
C68	0.6738 (4)	1.1006 (3)	0.2991 (3)	0.1278 (18)	
H68	0.6770	1.1617	0.2802	0.153*	
C69	0.6153 (3)	1.0692 (3)	0.3591 (2)	0.0833 (10)	
C70	0.6075 (3)	0.9797 (3)	0.3810 (2)	0.0950 (12)	
H70	0.5643	0.9567	0.4204	0.114*	
C71	0.6625 (3)	0.9227 (3)	0.3458 (2)	0.0841 (10)	
H71	0.6556	0.8620	0.3623	0.101*	

### Atomic displacement parameters (Å^2^)

**Table:**

	*U*^11^	*U*^22^	*U*^33^	*U*^12^	*U*^13^	*U*^23^
S1	0.0351 (3)	0.0425 (4)	0.0712 (5)	−0.0045 (3)	−0.0091 (3)	−0.0073 (3)
S2	0.0531 (4)	0.0521 (4)	0.0765 (5)	0.0083 (3)	−0.0126 (4)	−0.0308 (4)
S3	0.0511 (4)	0.0731 (5)	0.0756 (5)	−0.0196 (4)	0.0114 (4)	−0.0365 (4)
S4	0.0399 (4)	0.0493 (4)	0.0695 (5)	0.0021 (3)	0.0070 (3)	−0.0126 (3)
S5	0.0590 (11)	0.091 (3)	0.175 (4)	−0.0079 (15)	0.0024 (14)	−0.037 (3)
S6	0.135 (5)	0.135 (3)	0.0394 (16)	0.007 (3)	−0.019 (3)	−0.0198 (15)
S7	0.095 (3)	0.167 (5)	0.043 (2)	−0.012 (4)	0.0104 (16)	−0.031 (3)
S8	0.108 (2)	0.119 (3)	0.0641 (16)	0.021 (3)	−0.0073 (16)	−0.0442 (16)
C48	0.039 (3)	0.095 (4)	0.123 (5)	0.021 (3)	−0.015 (3)	−0.029 (3)
C56	0.137 (9)	0.165 (9)	0.080 (6)	−0.016 (7)	−0.008 (7)	−0.057 (6)
C64	0.134 (7)	0.149 (8)	0.054 (5)	−0.018 (7)	−0.003 (5)	0.008 (5)
C72	0.137 (7)	0.102 (6)	0.127 (6)	−0.001 (5)	0.010 (6)	−0.049 (5)
S5'	0.0595 (13)	0.092 (3)	0.175 (4)	−0.0095 (18)	0.0015 (16)	−0.037 (3)
S6'	0.135 (5)	0.177 (6)	0.056 (2)	−0.021 (4)	0.000 (3)	−0.029 (3)
S7'	0.122 (5)	0.152 (4)	0.057 (2)	0.019 (3)	0.024 (2)	−0.005 (2)
S8'	0.159 (6)	0.146 (6)	0.137 (5)	−0.001 (4)	0.007 (3)	−0.083 (4)
C48'	0.182 (10)	0.106 (8)	0.129 (9)	−0.016 (8)	0.038 (8)	−0.048 (7)
C56'	0.163 (10)	0.179 (9)	0.089 (7)	−0.013 (8)	−0.005 (8)	−0.064 (6)
C64'	0.173 (9)	0.127 (8)	0.106 (8)	−0.023 (7)	0.009 (6)	0.013 (6)
C72'	0.178 (14)	0.166 (13)	0.180 (13)	0.000 (9)	−0.003 (9)	−0.056 (9)
O1	0.0476 (11)	0.0799 (14)	0.0445 (11)	0.0093 (9)	−0.0129 (9)	−0.0113 (9)
O2	0.0602 (11)	0.0554 (11)	0.0418 (10)	−0.0062 (9)	−0.0020 (8)	−0.0172 (8)
O3	0.0394 (9)	0.0626 (11)	0.0409 (10)	−0.0054 (8)	0.0035 (8)	−0.0106 (8)
O4	0.0515 (11)	0.0623 (12)	0.0476 (11)	−0.0014 (9)	−0.0024 (9)	−0.0056 (9)
C1	0.0311 (12)	0.0451 (14)	0.0496 (15)	−0.0011 (10)	−0.0043 (11)	−0.0101 (11)
C2	0.0410 (14)	0.0534 (16)	0.0506 (16)	−0.0024 (12)	−0.0082 (12)	−0.0160 (12)
C3	0.0381 (14)	0.0607 (17)	0.0433 (14)	0.0025 (12)	−0.0055 (11)	−0.0117 (13)
C4	0.0439 (14)	0.0442 (15)	0.0536 (16)	0.0030 (12)	−0.0050 (12)	−0.0059 (12)
C5	0.0381 (13)	0.0429 (14)	0.0500 (15)	0.0031 (11)	−0.0071 (11)	−0.0143 (12)
C6	0.0313 (12)	0.0483 (15)	0.0429 (14)	0.0001 (11)	−0.0041 (10)	−0.0110 (11)
C7	0.0507 (16)	0.079 (2)	0.0449 (16)	0.0031 (15)	−0.0125 (13)	−0.0036 (14)
C8	0.126 (3)	0.105 (3)	0.066 (2)	−0.009 (2)	−0.021 (2)	0.012 (2)
C9	0.066 (2)	0.150 (4)	0.078 (2)	−0.014 (2)	−0.0329 (19)	0.004 (2)
C10	0.107 (3)	0.147 (4)	0.055 (2)	0.019 (3)	−0.009 (2)	−0.033 (2)
C11	0.0506 (15)	0.0406 (14)	0.0527 (16)	−0.0048 (12)	−0.0048 (13)	−0.0171 (12)
C12	0.0526 (16)	0.0503 (16)	0.0545 (16)	−0.0058 (13)	0.0058 (13)	−0.0213 (13)
C13	0.0592 (17)	0.0534 (16)	0.0524 (16)	−0.0065 (13)	−0.0007 (14)	−0.0210 (13)
C14	0.0511 (16)	0.0656 (18)	0.0531 (16)	−0.0071 (14)	−0.0069 (13)	−0.0242 (14)
C15	0.0513 (16)	0.0484 (15)	0.0513 (16)	−0.0064 (12)	0.0015 (13)	−0.0178 (12)
C16	0.0523 (16)	0.0412 (14)	0.0448 (15)	−0.0061 (12)	−0.0003 (12)	−0.0138 (11)
C17	0.074 (2)	0.085 (2)	0.0605 (19)	−0.0099 (18)	−0.0001 (17)	−0.0375 (17)
C18	0.094 (6)	0.100 (8)	0.046 (4)	−0.006 (7)	0.012 (4)	−0.030 (5)
C19	0.118 (6)	0.102 (5)	0.100 (5)	−0.004 (5)	−0.028 (5)	−0.065 (4)
C20	0.093 (5)	0.095 (6)	0.061 (5)	−0.002 (5)	−0.017 (4)	−0.029 (5)
C18'	0.102 (7)	0.110 (9)	0.083 (7)	−0.019 (7)	0.007 (5)	−0.041 (7)
C19'	0.143 (10)	0.117 (8)	0.101 (8)	−0.020 (8)	0.001 (8)	−0.071 (6)
C20'	0.096 (6)	0.108 (7)	0.069 (5)	−0.015 (6)	−0.019 (4)	−0.038 (5)
C21	0.0399 (14)	0.0571 (17)	0.0487 (15)	−0.0077 (12)	0.0011 (12)	−0.0156 (12)
C22	0.0465 (15)	0.0551 (17)	0.0519 (16)	−0.0114 (13)	0.0041 (13)	−0.0057 (13)
C23	0.0389 (14)	0.0682 (19)	0.0425 (14)	−0.0038 (13)	0.0006 (11)	−0.0105 (13)
C24	0.0389 (14)	0.0627 (18)	0.0449 (15)	0.0000 (12)	0.0012 (12)	−0.0172 (13)
C25	0.0329 (13)	0.0538 (16)	0.0464 (15)	−0.0013 (11)	−0.0017 (11)	−0.0107 (12)
C26	0.0306 (12)	0.0554 (16)	0.0407 (14)	−0.0043 (11)	0.0049 (11)	−0.0083 (12)
C27	0.0577 (18)	0.088 (2)	0.0436 (16)	−0.0090 (16)	0.0098 (14)	−0.0090 (15)
C28	0.075 (5)	0.090 (6)	0.051 (4)	−0.001 (5)	0.007 (4)	0.015 (4)
C29	0.143 (8)	0.148 (9)	0.070 (6)	−0.033 (7)	0.037 (6)	−0.033 (6)
C30	0.042 (3)	0.087 (6)	0.049 (4)	−0.005 (4)	0.010 (3)	0.003 (4)
C28'	0.118 (10)	0.100 (9)	0.084 (8)	−0.001 (7)	0.015 (7)	0.005 (6)
C29'	0.110 (7)	0.120 (7)	0.064 (5)	−0.028 (5)	0.030 (5)	−0.038 (5)
C30'	0.083 (7)	0.112 (9)	0.085 (7)	0.008 (7)	0.018 (5)	0.001 (6)
C31	0.0368 (13)	0.0401 (14)	0.0599 (17)	−0.0021 (11)	−0.0015 (12)	−0.0123 (12)
C32	0.0381 (14)	0.0443 (15)	0.0677 (18)	−0.0015 (12)	−0.0080 (13)	−0.0021 (13)
C33	0.0462 (15)	0.0424 (15)	0.0587 (17)	−0.0123 (12)	−0.0104 (13)	0.0012 (12)
C34	0.0380 (13)	0.0410 (14)	0.0570 (16)	−0.0083 (11)	0.0004 (12)	−0.0045 (12)
C35	0.0350 (13)	0.0339 (13)	0.0593 (16)	−0.0024 (10)	−0.0071 (12)	−0.0119 (12)
C36	0.0434 (14)	0.0355 (13)	0.0477 (15)	−0.0004 (11)	−0.0055 (12)	−0.0093 (11)
C37	0.0621 (19)	0.065 (2)	0.069 (2)	−0.0211 (16)	−0.0154 (16)	0.0133 (16)
C38	0.061 (4)	0.118 (6)	0.089 (5)	−0.018 (5)	0.000 (4)	0.039 (5)
C39	0.093 (5)	0.048 (3)	0.071 (4)	0.007 (3)	−0.021 (4)	0.007 (3)
C40	0.107 (7)	0.067 (4)	0.054 (4)	−0.006 (4)	−0.016 (4)	−0.004 (3)
C38'	0.095 (5)	0.107 (6)	0.061 (4)	0.005 (5)	−0.013 (4)	0.001 (4)
C39'	0.161 (7)	0.070 (5)	0.085 (5)	−0.021 (5)	0.000 (5)	0.012 (4)
C40'	0.065 (6)	0.108 (9)	0.089 (8)	−0.010 (6)	−0.021 (5)	0.015 (7)
C41	0.062 (2)	0.162 (4)	0.0470 (19)	−0.015 (2)	−0.0070 (16)	−0.011 (2)
C42	0.0555 (17)	0.071 (2)	0.0429 (15)	−0.0130 (15)	−0.0041 (13)	−0.0116 (14)
C43	0.071 (2)	0.069 (2)	0.083 (2)	0.0003 (18)	0.0100 (18)	−0.0301 (17)
C44	0.067 (2)	0.067 (2)	0.106 (3)	−0.0133 (17)	0.0039 (19)	−0.0374 (19)
C45	0.0644 (19)	0.0563 (19)	0.076 (2)	−0.0111 (16)	0.0012 (16)	−0.0114 (15)
C46	0.070 (2)	0.054 (2)	0.132 (3)	−0.0011 (17)	0.004 (2)	−0.030 (2)
C47	0.084 (3)	0.060 (2)	0.110 (3)	−0.0286 (19)	0.007 (2)	−0.0222 (19)
C49	0.099 (3)	0.061 (2)	0.0546 (18)	−0.0098 (17)	−0.0013 (17)	−0.0103 (15)
C50	0.0586 (17)	0.071 (2)	0.0445 (16)	−0.0104 (15)	−0.0009 (13)	−0.0068 (14)
C51	0.104 (3)	0.069 (2)	0.060 (2)	−0.0149 (19)	−0.0018 (19)	−0.0024 (17)
C52	0.101 (3)	0.103 (3)	0.050 (2)	−0.021 (2)	−0.0058 (18)	0.008 (2)
C53	0.076 (2)	0.107 (3)	0.0442 (18)	−0.013 (2)	−0.0002 (15)	−0.0153 (18)
C54	0.086 (2)	0.083 (2)	0.0519 (19)	−0.0030 (18)	−0.0057 (16)	−0.0177 (17)
C55	0.074 (2)	0.070 (2)	0.0438 (16)	0.0010 (16)	−0.0058 (14)	−0.0099 (14)
C57	0.0465 (16)	0.082 (2)	0.0534 (17)	0.0003 (15)	−0.0019 (13)	−0.0102 (15)
C58	0.0492 (16)	0.0563 (16)	0.0424 (15)	−0.0047 (13)	−0.0014 (12)	−0.0094 (12)
C59	0.0503 (17)	0.077 (2)	0.0464 (16)	0.0027 (14)	−0.0018 (13)	−0.0093 (14)
C60	0.0531 (17)	0.096 (2)	0.0527 (18)	0.0003 (16)	0.0019 (14)	−0.0193 (16)
C61	0.075 (2)	0.083 (2)	0.0451 (17)	−0.0056 (17)	−0.0005 (15)	−0.0204 (15)
C62	0.078 (2)	0.091 (2)	0.0457 (17)	−0.0085 (18)	−0.0139 (16)	−0.0142 (16)
C63	0.0510 (16)	0.074 (2)	0.0609 (19)	−0.0034 (14)	−0.0106 (14)	−0.0180 (15)
C65	0.0573 (18)	0.108 (3)	0.0528 (18)	0.0022 (18)	−0.0069 (15)	−0.0219 (17)
C66	0.066 (2)	0.087 (2)	0.0519 (18)	−0.0102 (18)	−0.0030 (15)	−0.0192 (17)
C67	0.159 (5)	0.109 (4)	0.104 (3)	−0.037 (3)	0.060 (3)	−0.031 (3)
C68	0.178 (5)	0.085 (3)	0.115 (4)	−0.014 (3)	0.039 (4)	−0.028 (3)
C69	0.092 (3)	0.099 (3)	0.066 (2)	0.002 (2)	−0.009 (2)	−0.036 (2)
C70	0.087 (3)	0.128 (4)	0.071 (2)	−0.010 (3)	0.018 (2)	−0.033 (2)
C71	0.093 (3)	0.085 (2)	0.072 (2)	−0.008 (2)	0.011 (2)	−0.0201 (19)

### Geometric parameters (Å, º)

**Table:**

S1—C1	1.768 (3)	C23—C24	1.378 (4)
S1—C35	1.779 (2)	C23—C27	1.526 (4)
S2—C11	1.770 (3)	C24—C25	1.390 (3)
S2—C5	1.777 (3)	C24—H24	0.9300
S3—C15	1.772 (3)	C25—C26	1.390 (4)
S3—C21	1.772 (3)	C27—C30'	1.445 (16)
S4—C25	1.773 (3)	C27—C28	1.454 (12)
S4—C31	1.773 (3)	C27—C29	1.510 (14)
S5—C45	1.762 (8)	C27—C29'	1.574 (12)
S5—C48	1.769 (8)	C27—C30	1.580 (10)
S6—C53	1.756 (9)	C27—C28'	1.683 (17)
S6—C56	1.816 (14)	C28—H28A	0.9600
S7—C64	1.800 (12)	C28—H28B	0.9600
S7—C61	1.811 (9)	C28—H28C	0.9600
S8—C69	1.773 (7)	C29—H29A	0.9600
S8—C72	1.791 (11)	C29—H29B	0.9600
C48—H48A	0.9600	C29—H29C	0.9600
C48—H48B	0.9600	C30—H30A	0.9600
C48—H48C	0.9600	C30—H30B	0.9600
C56—H56A	0.9600	C30—H30C	0.9600
C56—H56B	0.9600	C28'—H28D	0.9600
C56—H56C	0.9600	C28'—H28E	0.9600
C64—H64A	0.9600	C28'—H28F	0.9600
C64—H64B	0.9600	C29'—H29D	0.9600
C64—H64C	0.9600	C29'—H29E	0.9600
C72—H72D	0.9600	C29'—H29F	0.9600
C72—H72E	0.9600	C30'—H30D	0.9600
C72—H72F	0.9600	C30'—H30E	0.9600
S5'—C48'	1.792 (19)	C30'—H30F	0.9600
S5'—C45	1.827 (17)	C31—C32	1.380 (3)
S6'—C56'	1.740 (14)	C31—C36	1.405 (3)
S6'—C53	1.787 (10)	C32—C33	1.388 (4)
S7'—C61	1.734 (10)	C32—H32	0.9300
S7'—C64'	1.760 (13)	C33—C34	1.389 (3)
S8'—C69	1.756 (10)	C33—C37	1.532 (4)
S8'—C72'	1.812 (17)	C34—C35	1.387 (3)
C48'—H48D	0.9600	C34—H34	0.9300
C48'—H48E	0.9600	C35—C36	1.384 (3)
C48'—H48F	0.9600	C37—C38	1.416 (8)
C56'—H56D	0.9600	C37—C40'	1.423 (16)
C56'—H56E	0.9600	C37—C39'	1.437 (9)
C56'—H56F	0.9600	C37—C40	1.506 (11)
C64'—H64D	0.9600	C37—C39	1.678 (7)
C64'—H64E	0.9600	C37—C38'	1.733 (9)
C64'—H64F	0.9600	C38—H38A	0.9600
C72'—H72A	0.9600	C38—H38B	0.9600
C72'—H72B	0.9600	C38—H38C	0.9600
C72'—H72C	0.9600	C39—H39A	0.9600
O1—C6	1.370 (3)	C39—H39B	0.9600
O1—C41	1.375 (4)	C39—H39C	0.9600
O2—C16	1.371 (3)	C40—H40A	0.9600
O2—C49	1.421 (3)	C40—H40B	0.9600
O3—C26	1.372 (3)	C40—H40C	0.9600
O3—C57	1.433 (3)	C38'—H38D	0.9600
O4—C36	1.376 (3)	C38'—H38E	0.9600
O4—C65	1.440 (4)	C38'—H38F	0.9600
C1—C2	1.379 (3)	C39'—H39D	0.9600
C1—C6	1.400 (3)	C39'—H39E	0.9600
C2—C3	1.396 (4)	C39'—H39F	0.9600
C2—H2	0.9300	C40'—H40D	0.9600
C3—C4	1.373 (4)	C40'—H40E	0.9600
C3—C7	1.542 (4)	C40'—H40F	0.9600
C4—C5	1.387 (3)	C41—C42	1.482 (4)
C4—H4	0.9300	C41—H41A	0.9700
C5—C6	1.384 (3)	C41—H41B	0.9700
C7—C10	1.512 (5)	C42—C43	1.366 (4)
C7—C8	1.522 (5)	C42—C47	1.373 (4)
C7—C9	1.527 (4)	C43—C44	1.357 (4)
C8—H8A	0.9600	C43—H43	0.9300
C8—H8B	0.9600	C44—C45	1.345 (4)
C8—H8C	0.9600	C44—H44	0.9300
C9—H9A	0.9600	C45—C46	1.357 (4)
C9—H9B	0.9600	C46—C47	1.388 (5)
C9—H9C	0.9600	C46—H46	0.9300
C10—H10A	0.9600	C47—H47	0.9300
C10—H10B	0.9600	C49—C50	1.489 (4)
C10—H10C	0.9600	C49—H49A	0.9700
C11—C12	1.383 (4)	C49—H49B	0.9700
C11—C16	1.393 (4)	C50—C55	1.371 (4)
C12—C13	1.390 (4)	C50—C51	1.382 (4)
C12—H12	0.9300	C51—C52	1.374 (5)
C13—C14	1.378 (4)	C51—H51	0.9300
C13—C17	1.533 (4)	C52—C53	1.361 (5)
C14—C15	1.395 (4)	C52—H52	0.9300
C14—H14	0.9300	C53—C54	1.385 (5)
C15—C16	1.386 (4)	C54—C55	1.383 (4)
C17—C20'	1.457 (14)	C54—H54	0.9300
C17—C18'	1.46 (2)	C55—H55	0.9300
C17—C19	1.534 (11)	C57—C58	1.504 (4)
C17—C19'	1.566 (18)	C57—H57A	0.9700
C17—C18	1.592 (18)	C57—H57B	0.9700
C17—C20	1.624 (13)	C58—C59	1.375 (4)
C18—H18A	0.9600	C58—C63	1.386 (4)
C18—H18B	0.9600	C59—C60	1.372 (4)
C18—H18C	0.9600	C59—H59	0.9300
C19—H19A	0.9600	C60—C61	1.388 (4)
C19—H19B	0.9600	C60—H60	0.9300
C19—H19C	0.9600	C61—C62	1.380 (4)
C20—H20A	0.9600	C62—C63	1.361 (4)
C20—H20B	0.9600	C62—H62	0.9300
C20—H20C	0.9600	C63—H63	0.9300
C18'—H18D	0.9600	C65—C66	1.498 (4)
C18'—H18E	0.9600	C65—H65A	0.9700
C18'—H18F	0.9600	C65—H65B	0.9700
C19'—H19D	0.9600	C66—C67	1.354 (5)
C19'—H19E	0.9600	C66—C71	1.373 (5)
C19'—H19F	0.9600	C67—C68	1.402 (6)
C20'—H20D	0.9600	C67—H67	0.9300
C20'—H20E	0.9600	C68—C69	1.325 (6)
C20'—H20F	0.9600	C68—H68	0.9300
C21—C26	1.383 (4)	C69—C70	1.364 (5)
C21—C22	1.399 (4)	C70—C71	1.376 (5)
C22—C23	1.386 (4)	C70—H70	0.9300
C22—H22	0.9300	C71—H71	0.9300
			
C1—S1—C35	106.14 (11)	C27—C30—H30B	109.5
C11—S2—C5	108.71 (11)	C27—C30—H30C	109.5
C15—S3—C21	108.63 (12)	C27—C28'—H28D	109.5
C25—S4—C31	108.22 (11)	C27—C28'—H28E	109.5
C45—S5—C48	106.3 (4)	H28D—C28'—H28E	109.5
C53—S6—C56	100.8 (7)	C27—C28'—H28F	109.5
C64—S7—C61	100.0 (6)	H28D—C28'—H28F	109.5
C69—S8—C72	102.6 (6)	H28E—C28'—H28F	109.5
C48'—S5'—C45	82.1 (9)	C27—C29'—H29D	109.5
C56'—S6'—C53	102.5 (8)	C27—C29'—H29E	109.5
C61—S7'—C64'	107.7 (8)	H29D—C29'—H29E	109.5
C69—S8'—C72'	107.6 (11)	C27—C29'—H29F	109.5
S5'—C48'—H48D	109.5	H29D—C29'—H29F	109.5
S5'—C48'—H48E	109.5	H29E—C29'—H29F	109.5
H48D—C48'—H48E	109.5	C27—C30'—H30D	109.5
S5'—C48'—H48F	109.5	C27—C30'—H30E	109.5
H48D—C48'—H48F	109.5	H30D—C30'—H30E	109.5
H48E—C48'—H48F	109.5	C27—C30'—H30F	109.5
S6'—C56'—H56D	109.5	H30D—C30'—H30F	109.5
S6'—C56'—H56E	109.5	H30E—C30'—H30F	109.5
H56D—C56'—H56E	109.5	C32—C31—C36	120.3 (2)
S6'—C56'—H56F	109.5	C32—C31—S4	117.03 (18)
H56D—C56'—H56F	109.5	C36—C31—S4	122.17 (19)
H56E—C56'—H56F	109.5	C31—C32—C33	122.4 (2)
S7'—C64'—H64D	109.5	C31—C32—H32	118.8
S7'—C64'—H64E	109.5	C33—C32—H32	118.8
H64D—C64'—H64E	109.5	C32—C33—C34	116.4 (2)
S7'—C64'—H64F	109.5	C32—C33—C37	121.2 (2)
H64D—C64'—H64F	109.5	C34—C33—C37	122.5 (2)
H64E—C64'—H64F	109.5	C35—C34—C33	122.4 (2)
S8'—C72'—H72A	109.5	C35—C34—H34	118.8
S8'—C72'—H72B	109.5	C33—C34—H34	118.8
H72A—C72'—H72B	109.5	C36—C35—C34	120.5 (2)
S8'—C72'—H72C	109.5	C36—C35—S1	124.13 (19)
H72A—C72'—H72C	109.5	C34—C35—S1	115.11 (18)
H72B—C72'—H72C	109.5	O4—C36—C35	123.7 (2)
C6—O1—C41	124.3 (2)	O4—C36—C31	118.2 (2)
C16—O2—C49	114.5 (2)	C35—C36—C31	117.9 (2)
C26—O3—C57	113.88 (18)	C38—C37—C40'	128.2 (8)
C36—O4—C65	116.4 (2)	C38—C37—C39'	56.3 (6)
C2—C1—C6	120.1 (2)	C40'—C37—C39'	117.1 (7)
C2—C1—S1	117.5 (2)	C38—C37—C40	117.8 (7)
C6—C1—S1	122.04 (19)	C40'—C37—C40	25.5 (7)
C1—C2—C3	122.3 (2)	C39'—C37—C40	135.4 (5)
C1—C2—H2	118.8	C38—C37—C33	115.0 (4)
C3—C2—H2	118.8	C40'—C37—C33	114.3 (7)
C4—C3—C2	116.4 (2)	C39'—C37—C33	110.9 (4)
C4—C3—C7	123.2 (2)	C40—C37—C33	110.3 (4)
C2—C3—C7	120.3 (3)	C38—C37—C39	106.5 (6)
C3—C4—C5	122.6 (2)	C40'—C37—C39	74.4 (6)
C3—C4—H4	118.7	C39'—C37—C39	52.7 (5)
C5—C4—H4	118.7	C40—C37—C39	99.8 (4)
C6—C5—C4	120.4 (2)	C33—C37—C39	105.5 (3)
C6—C5—S2	123.38 (19)	C38—C37—C38'	45.7 (5)
C4—C5—S2	115.79 (18)	C40'—C37—C38'	105.0 (7)
O1—C6—C5	122.5 (2)	C39'—C37—C38'	101.7 (6)
O1—C6—C1	119.2 (2)	C40—C37—C38'	82.6 (5)
C5—C6—C1	118.0 (2)	C33—C37—C38'	106.2 (3)
C10—C7—C8	107.3 (3)	C39—C37—C38'	145.1 (4)
C10—C7—C9	110.5 (3)	C37—C38—H38A	109.5
C8—C7—C9	108.6 (3)	C37—C38—H38B	109.5
C10—C7—C3	110.8 (2)	C37—C38—H38C	109.5
C8—C7—C3	111.5 (3)	C37—C39—H39A	109.5
C9—C7—C3	108.1 (2)	C37—C39—H39B	109.5
C7—C8—H8A	109.5	C37—C39—H39C	109.5
C7—C8—H8B	109.5	C37—C40—H40A	109.5
H8A—C8—H8B	109.5	C37—C40—H40B	109.5
C7—C8—H8C	109.5	C37—C40—H40C	109.5
H8A—C8—H8C	109.5	C37—C38'—H38D	109.5
H8B—C8—H8C	109.5	C37—C38'—H38E	109.5
C7—C9—H9A	109.5	H38D—C38'—H38E	109.5
C7—C9—H9B	109.5	C37—C38'—H38F	109.5
H9A—C9—H9B	109.5	H38D—C38'—H38F	109.5
C7—C9—H9C	109.5	H38E—C38'—H38F	109.5
H9A—C9—H9C	109.5	C37—C39'—H39D	109.5
H9B—C9—H9C	109.5	C37—C39'—H39E	109.5
C7—C10—H10A	109.5	H39D—C39'—H39E	109.5
C7—C10—H10B	109.5	C37—C39'—H39F	109.5
H10A—C10—H10B	109.5	H39D—C39'—H39F	109.5
C7—C10—H10C	109.5	H39E—C39'—H39F	109.5
H10A—C10—H10C	109.5	C37—C40'—H40D	109.5
H10B—C10—H10C	109.5	C37—C40'—H40E	109.5
C12—C11—C16	119.9 (2)	H40D—C40'—H40E	109.5
C12—C11—S2	116.2 (2)	C37—C40'—H40F	109.5
C16—C11—S2	123.2 (2)	H40D—C40'—H40F	109.5
C11—C12—C13	122.3 (2)	H40E—C40'—H40F	109.5
C11—C12—H12	118.8	O1—C41—C42	120.6 (3)
C13—C12—H12	118.8	O1—C41—H41A	107.2
C14—C13—C12	116.9 (2)	C42—C41—H41A	107.2
C14—C13—C17	120.9 (3)	O1—C41—H41B	107.2
C12—C13—C17	122.2 (3)	C42—C41—H41B	107.2
C13—C14—C15	122.1 (3)	H41A—C41—H41B	106.8
C13—C14—H14	119.0	C43—C42—C47	115.1 (3)
C15—C14—H14	119.0	C43—C42—C41	123.2 (3)
C16—C15—C14	120.1 (2)	C47—C42—C41	121.6 (3)
C16—C15—S3	124.2 (2)	C44—C43—C42	123.4 (3)
C14—C15—S3	115.2 (2)	C44—C43—H43	118.3
O2—C16—C15	120.7 (2)	C42—C43—H43	118.3
O2—C16—C11	120.6 (2)	C45—C44—C43	120.4 (3)
C15—C16—C11	118.7 (2)	C45—C44—H44	119.8
C20'—C17—C18'	117.9 (10)	C43—C44—H44	119.8
C20'—C17—C13	114.7 (6)	C44—C45—C46	119.1 (3)
C18'—C17—C13	115.0 (8)	C44—C45—S5	115.1 (3)
C20'—C17—C19	89.3 (6)	C46—C45—S5	125.7 (3)
C18'—C17—C19	108.1 (7)	C44—C45—S5'	122.0 (6)
C13—C17—C19	108.0 (5)	C46—C45—S5'	118.5 (6)
C20'—C17—C19'	114.6 (8)	S5—C45—S5'	10.0 (10)
C18'—C17—C19'	86.7 (8)	C45—C46—C47	119.6 (3)
C13—C17—C19'	103.6 (7)	C45—C46—H46	120.2
C19—C17—C19'	27.2 (6)	C47—C46—H46	120.2
C20'—C17—C18	110.2 (9)	C42—C47—C46	122.2 (3)
C18'—C17—C18	18.6 (9)	C42—C47—H47	118.9
C13—C17—C18	107.7 (7)	C46—C47—H47	118.9
C19—C17—C18	126.3 (7)	O2—C49—C50	109.5 (3)
C19'—C17—C18	105.3 (9)	O2—C49—H49A	109.8
C20'—C17—C20	20.8 (7)	C50—C49—H49A	109.8
C18'—C17—C20	107.1 (9)	O2—C49—H49B	109.8
C13—C17—C20	108.8 (5)	C50—C49—H49B	109.8
C19—C17—C20	109.9 (6)	H49A—C49—H49B	108.2
C19'—C17—C20	134.2 (8)	C55—C50—C51	117.9 (3)
C18—C17—C20	94.8 (9)	C55—C50—C49	123.2 (3)
C17—C18—H18A	109.5	C51—C50—C49	118.9 (3)
C17—C18—H18B	109.5	C52—C51—C50	121.2 (4)
C17—C18—H18C	109.5	C52—C51—H51	119.4
C17—C19—H19A	109.5	C50—C51—H51	119.4
C17—C19—H19B	109.5	C53—C52—C51	121.2 (3)
C17—C19—H19C	109.5	C53—C52—H52	119.4
C17—C20—H20A	109.5	C51—C52—H52	119.4
C17—C20—H20B	109.5	C52—C53—C54	118.2 (3)
C17—C20—H20C	109.5	C52—C53—S6	114.4 (4)
C17—C18'—H18D	109.5	C54—C53—S6	126.9 (5)
C17—C18'—H18E	109.5	C52—C53—S6'	120.3 (5)
H18D—C18'—H18E	109.5	C54—C53—S6'	120.9 (5)
C17—C18'—H18F	109.5	S6—C53—S6'	15.5 (5)
H18D—C18'—H18F	109.5	C55—C54—C53	120.7 (3)
H18E—C18'—H18F	109.5	C55—C54—H54	119.7
C17—C19'—H19D	109.5	C53—C54—H54	119.7
C17—C19'—H19E	109.5	C50—C55—C54	120.9 (3)
H19D—C19'—H19E	109.5	C50—C55—H55	119.5
C17—C19'—H19F	109.5	C54—C55—H55	119.5
H19D—C19'—H19F	109.5	O3—C57—C58	109.3 (2)
H19E—C19'—H19F	109.5	O3—C57—H57A	109.8
C17—C20'—H20D	109.5	C58—C57—H57A	109.8
C17—C20'—H20E	109.5	O3—C57—H57B	109.8
H20D—C20'—H20E	109.5	C58—C57—H57B	109.8
C17—C20'—H20F	109.5	H57A—C57—H57B	108.3
H20D—C20'—H20F	109.5	C59—C58—C63	118.2 (2)
H20E—C20'—H20F	109.5	C59—C58—C57	123.4 (2)
C26—C21—C22	120.6 (2)	C63—C58—C57	118.4 (2)
C26—C21—S3	123.6 (2)	C60—C59—C58	120.4 (3)
C22—C21—S3	115.3 (2)	C60—C59—H59	119.8
C23—C22—C21	121.2 (3)	C58—C59—H59	119.8
C23—C22—H22	119.4	C59—C60—C61	121.2 (3)
C21—C22—H22	119.4	C59—C60—H60	119.4
C24—C23—C22	117.2 (2)	C61—C60—H60	119.4
C24—C23—C27	122.4 (3)	C62—C61—C60	118.1 (3)
C22—C23—C27	120.4 (3)	C62—C61—S7'	120.1 (4)
C23—C24—C25	122.7 (3)	C60—C61—S7'	121.8 (4)
C23—C24—H24	118.7	C62—C61—S7	128.9 (4)
C25—C24—H24	118.7	C60—C61—S7	112.9 (4)
C26—C25—C24	119.6 (2)	S7'—C61—S7	10.2 (7)
C26—C25—S4	123.81 (19)	C63—C62—C61	120.4 (3)
C24—C25—S4	115.7 (2)	C63—C62—H62	119.8
O3—C26—C21	120.5 (2)	C61—C62—H62	119.8
O3—C26—C25	120.8 (2)	C62—C63—C58	121.6 (3)
C21—C26—C25	118.7 (2)	C62—C63—H63	119.2
C30'—C27—C28	124.7 (8)	C58—C63—H63	119.2
C30'—C27—C29	108.4 (8)	O4—C65—C66	114.4 (2)
C28—C27—C29	85.6 (7)	O4—C65—H65A	108.7
C30'—C27—C23	111.6 (7)	C66—C65—H65A	108.7
C28—C27—C23	112.3 (5)	O4—C65—H65B	108.7
C29—C27—C23	110.4 (5)	C66—C65—H65B	108.7
C30'—C27—C29'	79.6 (7)	H65A—C65—H65B	107.6
C28—C27—C29'	112.4 (7)	C67—C66—C71	115.9 (3)
C29—C27—C29'	31.4 (5)	C67—C66—C65	122.3 (3)
C23—C27—C29'	111.6 (5)	C71—C66—C65	121.7 (3)
C30'—C27—C30	25.2 (6)	C66—C67—C68	122.2 (4)
C28—C27—C30	108.9 (6)	C66—C67—H67	118.9
C29—C27—C30	130.7 (6)	C68—C67—H67	118.9
C23—C27—C30	106.8 (4)	C69—C68—C67	120.8 (4)
C29'—C27—C30	104.2 (6)	C69—C68—H68	119.6
C30'—C27—C28'	110.4 (9)	C67—C68—H68	119.6
C28—C27—C28'	21.6 (9)	C68—C69—C70	118.0 (4)
C29—C27—C28'	106.3 (8)	C68—C69—S8'	117.1 (6)
C23—C27—C28'	109.6 (7)	C70—C69—S8'	124.8 (5)
C29'—C27—C28'	129.6 (8)	C68—C69—S8	129.1 (5)
C30—C27—C28'	90.2 (7)	C70—C69—S8	112.8 (4)
C27—C28—H28A	109.5	S8'—C69—S8	12.7 (7)
C27—C28—H28B	109.5	C69—C70—C71	121.3 (4)
C27—C28—H28C	109.5	C69—C70—H70	119.4
C27—C29—H29A	109.5	C71—C70—H70	119.4
C27—C29—H29B	109.5	C66—C71—C70	121.6 (4)
C27—C29—H29C	109.5	C66—C71—H71	119.2
C27—C30—H30A	109.5	C70—C71—H71	119.2
			
C35—S1—C1—C2	−128.12 (19)	C1—S1—C35—C34	−133.1 (2)
C35—S1—C1—C6	58.3 (2)	C65—O4—C36—C35	70.1 (3)
C6—C1—C2—C3	−1.1 (4)	C65—O4—C36—C31	−115.1 (3)
S1—C1—C2—C3	−174.75 (19)	C34—C35—C36—O4	178.6 (2)
C1—C2—C3—C4	−1.7 (4)	S1—C35—C36—O4	−7.8 (4)
C1—C2—C3—C7	179.2 (2)	C34—C35—C36—C31	3.8 (4)
C2—C3—C4—C5	2.2 (4)	S1—C35—C36—C31	177.35 (19)
C7—C3—C4—C5	−178.7 (2)	C32—C31—C36—O4	−178.8 (2)
C3—C4—C5—C6	0.0 (4)	S4—C31—C36—O4	9.2 (3)
C3—C4—C5—S2	172.9 (2)	C32—C31—C36—C35	−3.7 (4)
C11—S2—C5—C6	−56.4 (2)	S4—C31—C36—C35	−175.69 (19)
C11—S2—C5—C4	130.9 (2)	C32—C33—C37—C38	168.4 (7)
C41—O1—C6—C5	−79.4 (4)	C34—C33—C37—C38	−11.2 (8)
C41—O1—C6—C1	106.7 (3)	C32—C33—C37—C40'	−28.2 (8)
C4—C5—C6—O1	−176.8 (2)	C34—C33—C37—C40'	152.3 (7)
S2—C5—C6—O1	10.8 (3)	C32—C33—C37—C39'	106.8 (6)
C4—C5—C6—C1	−2.7 (3)	C34—C33—C37—C39'	−72.7 (7)
S2—C5—C6—C1	−175.14 (18)	C32—C33—C37—C40	−55.5 (5)
C2—C1—C6—O1	177.5 (2)	C34—C33—C37—C40	125.0 (5)
S1—C1—C6—O1	−9.1 (3)	C32—C33—C37—C39	51.4 (4)
C2—C1—C6—C5	3.3 (3)	C34—C33—C37—C39	−128.1 (4)
S1—C1—C6—C5	176.64 (17)	C32—C33—C37—C38'	−143.4 (4)
C4—C3—C7—C10	−127.8 (3)	C34—C33—C37—C38'	37.0 (5)
C2—C3—C7—C10	51.3 (4)	C6—O1—C41—C42	−21.9 (5)
C4—C3—C7—C8	−8.4 (4)	O1—C41—C42—C43	−80.9 (5)
C2—C3—C7—C8	170.7 (3)	O1—C41—C42—C47	102.2 (4)
C4—C3—C7—C9	110.9 (3)	C47—C42—C43—C44	3.1 (5)
C2—C3—C7—C9	−70.0 (3)	C41—C42—C43—C44	−174.0 (3)
C5—S2—C11—C12	139.5 (2)	C42—C43—C44—C45	−2.3 (6)
C5—S2—C11—C16	−49.6 (2)	C43—C44—C45—C46	0.8 (5)
C16—C11—C12—C13	−0.2 (4)	C43—C44—C45—S5	179.0 (5)
S2—C11—C12—C13	171.0 (2)	C43—C44—C45—S5'	−172.7 (11)
C11—C12—C13—C14	1.1 (4)	C48—S5—C45—C44	−177.8 (5)
C11—C12—C13—C17	−177.4 (3)	C48—S5—C45—C46	0.4 (8)
C12—C13—C14—C15	−1.5 (4)	C48—S5—C45—S5'	47 (6)
C17—C13—C14—C15	177.0 (3)	C48'—S5'—C45—C44	−153.6 (7)
C13—C14—C15—C16	0.9 (4)	C48'—S5'—C45—C46	32.9 (13)
C13—C14—C15—S3	−171.2 (2)	C48'—S5'—C45—S5	−105 (7)
C21—S3—C15—C16	46.1 (3)	C44—C45—C46—C47	−0.3 (6)
C21—S3—C15—C14	−142.2 (2)	S5—C45—C46—C47	−178.3 (5)
C49—O2—C16—C15	89.0 (3)	S5'—C45—C46—C47	173.4 (11)
C49—O2—C16—C11	−92.3 (3)	C43—C42—C47—C46	−2.6 (5)
C14—C15—C16—O2	178.7 (2)	C41—C42—C47—C46	174.6 (3)
S3—C15—C16—O2	−9.9 (4)	C45—C46—C47—C42	1.3 (6)
C14—C15—C16—C11	0.0 (4)	C16—O2—C49—C50	−177.7 (2)
S3—C15—C16—C11	171.4 (2)	O2—C49—C50—C55	7.8 (4)
C12—C11—C16—O2	−179.1 (2)	O2—C49—C50—C51	−173.5 (3)
S2—C11—C16—O2	10.3 (3)	C55—C50—C51—C52	−0.6 (5)
C12—C11—C16—C15	−0.3 (4)	C49—C50—C51—C52	−179.4 (3)
S2—C11—C16—C15	−170.97 (19)	C50—C51—C52—C53	1.8 (6)
C14—C13—C17—C20'	17.0 (7)	C51—C52—C53—C54	−2.1 (6)
C12—C13—C17—C20'	−164.6 (6)	C51—C52—C53—S6	−174.7 (4)
C14—C13—C17—C18'	158.5 (7)	C51—C52—C53—S6'	169.2 (4)
C12—C13—C17—C18'	−23.1 (8)	C56—S6—C53—C52	−173.0 (5)
C14—C13—C17—C19	−80.8 (5)	C56—S6—C53—C54	15.2 (8)
C12—C13—C17—C19	97.6 (5)	C56—S6—C53—S6'	−57 (3)
C14—C13—C17—C19'	−108.7 (7)	C56'—S6'—C53—C52	150.9 (6)
C12—C13—C17—C19'	69.7 (7)	C56'—S6'—C53—C54	−38.0 (8)
C14—C13—C17—C18	140.1 (7)	C56'—S6'—C53—S6	79 (3)
C12—C13—C17—C18	−41.5 (7)	C52—C53—C54—C55	1.4 (5)
C14—C13—C17—C20	38.5 (6)	S6—C53—C54—C55	172.9 (4)
C12—C13—C17—C20	−143.1 (6)	S6'—C53—C54—C55	−169.8 (4)
C15—S3—C21—C26	49.1 (2)	C51—C50—C55—C54	−0.1 (5)
C15—S3—C21—C22	−138.9 (2)	C49—C50—C55—C54	178.6 (3)
C26—C21—C22—C23	−0.2 (4)	C53—C54—C55—C50	−0.3 (5)
S3—C21—C22—C23	−172.4 (2)	C26—O3—C57—C58	−173.4 (2)
C21—C22—C23—C24	−0.7 (4)	O3—C57—C58—C59	1.3 (4)
C21—C22—C23—C27	177.2 (2)	O3—C57—C58—C63	−177.6 (2)
C22—C23—C24—C25	1.0 (4)	C63—C58—C59—C60	1.1 (5)
C27—C23—C24—C25	−176.8 (2)	C57—C58—C59—C60	−177.8 (3)
C23—C24—C25—C26	−0.6 (4)	C58—C59—C60—C61	0.7 (5)
C23—C24—C25—S4	169.39 (19)	C59—C60—C61—C62	−2.4 (5)
C31—S4—C25—C26	−47.7 (2)	C59—C60—C61—S7'	175.8 (5)
C31—S4—C25—C24	142.83 (19)	C59—C60—C61—S7	−178.5 (4)
C57—O3—C26—C21	89.2 (3)	C64'—S7'—C61—C62	38.4 (9)
C57—O3—C26—C25	−92.4 (3)	C64'—S7'—C61—C60	−139.8 (7)
C22—C21—C26—O3	179.1 (2)	C64'—S7'—C61—S7	−171 (4)
S3—C21—C26—O3	−9.3 (3)	C64—S7—C61—C62	15.1 (9)
C22—C21—C26—C25	0.7 (4)	C64—S7—C61—C60	−169.4 (5)
S3—C21—C26—C25	172.24 (18)	C64—S7—C61—S7'	−17 (3)
C24—C25—C26—O3	−178.8 (2)	C60—C61—C62—C63	2.3 (5)
S4—C25—C26—O3	12.1 (3)	S7'—C61—C62—C63	−176.0 (5)
C24—C25—C26—C21	−0.3 (4)	S7—C61—C62—C63	177.6 (5)
S4—C25—C26—C21	−169.42 (18)	C61—C62—C63—C58	−0.4 (5)
C24—C23—C27—C30'	80.8 (7)	C59—C58—C63—C62	−1.3 (5)
C22—C23—C27—C30'	−97.1 (7)	C57—C58—C63—C62	177.7 (3)
C24—C23—C27—C28	−133.6 (6)	C36—O4—C65—C66	81.3 (3)
C22—C23—C27—C28	48.6 (6)	O4—C65—C66—C67	−99.8 (4)
C24—C23—C27—C29	−39.9 (6)	O4—C65—C66—C71	81.0 (4)
C22—C23—C27—C29	142.3 (6)	C71—C66—C67—C68	1.7 (7)
C24—C23—C27—C29'	−6.3 (6)	C65—C66—C67—C68	−177.6 (4)
C22—C23—C27—C29'	175.9 (5)	C66—C67—C68—C69	2.2 (8)
C24—C23—C27—C30	107.0 (5)	C67—C68—C69—C70	−5.1 (7)
C22—C23—C27—C30	−70.8 (5)	C67—C68—C69—S8'	172.1 (6)
C24—C23—C27—C28'	−156.6 (7)	C67—C68—C69—S8	177.0 (5)
C22—C23—C27—C28'	25.6 (7)	C72'—S8'—C69—C68	15.4 (12)
C25—S4—C31—C32	128.5 (2)	C72'—S8'—C69—C70	−167.7 (10)
C25—S4—C31—C36	−59.2 (2)	C72'—S8'—C69—S8	−147 (3)
C36—C31—C32—C33	0.4 (4)	C72—S8—C69—C68	19.2 (8)
S4—C31—C32—C33	172.8 (2)	C72—S8—C69—C70	−158.8 (6)
C31—C32—C33—C34	2.8 (4)	C72—S8—C69—S8'	39 (2)
C31—C32—C33—C37	−176.8 (3)	C68—C69—C70—C71	4.2 (6)
C32—C33—C34—C35	−2.7 (4)	S8'—C69—C70—C71	−172.7 (5)
C37—C33—C34—C35	176.9 (3)	S8—C69—C70—C71	−177.6 (4)
C33—C34—C35—C36	−0.6 (4)	C67—C66—C71—C70	−2.6 (6)
C33—C34—C35—S1	−174.7 (2)	C65—C66—C71—C70	176.7 (3)
C1—S1—C35—C36	53.0 (2)	C69—C70—C71—C66	−0.3 (6)
